# Common Factors in Shoulder and Hip Arthroplasty Implant Failures: A Historical Review

**DOI:** 10.3390/jcm13082370

**Published:** 2024-04-18

**Authors:** Reed Andrews, Josué G. Layuno-Matos, Mark A. Frankle

**Affiliations:** 1 Department of Orthopaedics, School of Medicine, University of South Florida, Tampa, FL 33612, USA; andrewsr1@usf.edu; 2 Foundation for Orthopaedic Research and Education, Tampa, FL 33607, USA; jlayuno@foreonline.org; 3 Florida Orthopaedic Institute, Tampa, FL 33637, USA

**Keywords:** hip arthroplasty, shoulder arthroplasty, history of arthroplasty, total hip arthroplasty, THA, total shoulder arthroplasty, TSA, failure, hemiarthroplasty, revision

## Abstract

In this era of subspecialty care in orthopedics, iterations of implant design can occur in a silo which then precludes gaining knowledge from failures of implant design that may have occurred in different subspecialties. This literature review describes the history of failures in hip and shoulder arthroplasties with the purpose of identifying similar factors that led to previous implant failures. A review of the literature was performed by two reviewers assessing articles that described failed hip and shoulder arthroplasty systems over time. We identified and analyzed 53 implant failures—23 in hip arthroplasty and 30 in shoulder arthroplasty. These failures were categorized as material, mechanical, and technical. In hip arthroplasty, 48% were material, 39% mechanical, and 13% technical failures. In shoulder arthroplasty, the distribution was 10% material, 70% mechanical, and 20% technical failures. The distribution of these failures highlights similar and sometimes repeated failure mechanisms between subspecialties. This accentuates the importance of a collaborative approach to improve future arthroplasty designs.

## 1. Introduction

Arthroplasty has revolutionized the landscape of orthopedic surgery, providing effective solutions for enhancing mobility and alleviating pain in patients with debilitating shoulder and hip conditions. The evolution of shoulder and hip arthroplasties has been marked by continuous advancements in surgical techniques, materials, and implant designs. Despite these strides, implant failures remain a concern, prompting the need for a comprehensive examination to better understand the underlying causes and to establish criteria for implant success.

The history of hip arthroplasty has progressed significantly over the last century and a half with many different successes and failures along the way. It began in the early 19th century with Dr. Gluck’s ivory hemi hip replacement (1880s) and has since gone through key implant designs such as the Smith-Petersen mold arthroplasty (1923), McKee-Farrar total hip arthroplasty (THA) (1960), and the low friction THA by Dr. John Charnley (1962) to reach the systems we use today [1,2,3]. Although many of these implants demonstrated successful outcomes, our goal was to identify described failure mechanisms that were improved upon in future designs and techniques, regardless of their incidence.

Similarly, the history of shoulder arthroplasty has gone through many stages of development. Since its inception in 1893 with Dr. Jules Emile Péan’s ivory total shoulder, shoulder arthroplasty has seen changes in materials, designs, and geometries that have led to the modern implants used today [4,5,6].

The literature has shown that some developments in shoulder arthroplasty have been influenced by similar advancements in hip arthroplasty such is the case of Dr. Péan’s first shoulder arthroplasty, which utilized ivory implants, and Dr. Charles Neer’s hemiarthroplasty (HA) in 1951 that was likely inspired by the success of HA for the treatment of hip fractures [4,6,7].

This literature review’s main objective is to identify common factors contributing to implant failures across shoulder and hip arthroplasties. By analyzing material, mechanical, and technical aspects as to why different prostheses failed, we aim to better understand how we arrived at implant designs that are currently in use and how developments in hip and shoulder arthroplasties have influenced their respective iterations. With this, we intend to create the first applicable criteria that classify failure methods based on the heuristic surgeons use to group failed arthroplasties. We also aim to emphasize the importance of communication amongst subspecialties during implant design processes, to optimize patient outcomes and avoid preventable failures for those performing shoulder and hip arthroplasties.

## 2. Materials and Methods

A review of the literature was conducted to elucidate the historical contexts, trends, and modes of failure associated with shoulder and hip arthroplasties. Articles were searched across different search engines and databases such as PubMed, Scopus, and Embase. Included studies provided historical insights into the evolution of designs, progressions in surgical techniques, and outcomes of shoulder and hip arthroplasties, with a specific focus on implant failures and complications. Once an article was identified to have pertinent material, its reference section was also reviewed for additional articles.

The search was not restricted by publication date in order to provide a broad historical overview. Two researchers (RA and JL) independently screened titles and abstracts, followed by a full-text assessment of selected articles to determine final inclusion. Data extraction involved retrieving publication details, historical contexts, and relevant outcomes.

All included failed prostheses were subcategorized based on their respective common failure modes into one of three broad failure categories: material, mechanical, or technical. Failure designation was decided by the initial reviewer (RA) and approved by an additional reviewer (MAF). Material failure was defined as a failure/wear of the implant materials (i.e., polyethylene wear, bearing surface wear particles, polytetrafluoroethylene (PTFE) wear, and foreign body reactions) that was deemed the primary cause of construct failure. Mechanical failure was defined as a violation of a biomechanical principle deemed the primary cause of construct failure (i.e., scapular notching and glenoid component failure secondary to prosthetic impingement). Technical failure was defined by failure due to surgical technique (i.e., greater tuberosity osteotomy, greater trochanteric osteotomy, and approach) that was deemed the primary cause of construct failure. Once classified, the total of implant failures by subcategory and their breakdowns in their respective subspecialties were calculated. The synthesized data aimed to provide a simplified overview of the historical trajectory and commonalities in failure modes associated with shoulder and hip arthroplasty implants.

## 3. Results

A total of 32 articles met the inclusion criteria; of which, 53 implant failure modes were identified. These 53 implant failure modes consisted of 23 failures in hip arthroplasty and 30 failures in shoulder arthroplasty.

### 3.1. Implant Failures by Arthroplasty Type

1. Combined Hip and Shoulder Implant Failures:
  - 26% (14/53) were material failures;
  - 57% (30/53) were mechanical failures;
  - 17% (9/53) were technical failures.
2. Hip Arthroplasty Implant Failures:
  - 48% (11/23) were material failures;
  - 39% (9/23) were mechanical failures;
  - 13% (3/23) were technical failures.
3. Shoulder Arthroplasty Implant Failures:
  - 10% (3/30) were material failures;
  - 70% (21/30) were mechanical failures;
  - 2% (6/30) were technical failures.

### 3.2. Implant Failures by Category

#### 3.2.1. Material Failures

Material failures in hip arthroplasty include the use of ivory, acrylic, glass, polytetrafluoroethylene (PTFE), polyethylene, stainless steel, polyacetal, ceramic, and metal-on-metal (MoM). The ivory arthroplasty by Gluck (1880) faced failure due to a foreign body reaction and infection [1,8]. Smith-Petersen mold/cup arthroplasty (1923) experienced glass breakage during gait, while other molds lacked inert and durable properties [1,2]. Judet’s acrylic THA (1948) failures were the most common due to wear and/or fracture of the prosthesis and bone absorption [8,9]. In the case of Charnley’s early PTFE THA (~1955), the implant failed due to early PTFE wear leading to aseptic loosening and osteolysis. These complications prompted the development of ultrahigh-molecular-weight polyethylene (UHMWPE). Amidst the 1960s, Dr. Charnley popularized polymethylmethacrylate (PMMA) or bone cement, which became a milestone in orthopedics [3,8,10]. Charnley cemented metal-on-polyethylene (MoP) THA (1970) suffered from aseptic loosening and osteolysis attributed to polyethylene wear particles and “cement disease” [1,2,8,11]. Polyacetal femoral heads (1971) faced failure due to screw or glue fixation of the femoral head (1972), resulting in inadequate fixation and subsequent head/neck implant fracture [11,12]. Stainless steel heads were largely replaced by alumina and zirconia ceramic heads (1974) to avoid the concern of increased wear particle production [11]. Ceramic-on-ceramic (Alumina and Zirconia, 1985) faced problems such as chipping, squeaking, and fracture [1,11]. Stainless steel femoral stems had some reports of stress fractures and aseptic loosening that required revision and led to multiple iterations [12,13,14]. The third generation, large diameter metal-on-metal bearing surfaces (1988) resulted in metallosis, a carcinogenic condition, hypersensitivity reactions, and aseptic loosening [2,15]. A breakdown of failed hip implants attributed to material failures is listed in Table 1.

Material failures in shoulder arthroplasty include the use of acrylic and polyamide components. In the early 1950s, multiple surgeons including Krueger, deAnquin, Richards, and Judet all used acrylic components in their prostheses that failed due to a lack of durability, the production of wear particles, foreign body reactions, and subsequent component loosening [6,16,17]. MacAusland used a polyamide prosthesis that failed for the same reasons [17]. Refer to Table 2 for the distribution of material failures in shoulder arthroplasty.

#### 3.2.2. Mechanical Failures

Mechanical failures include acetabular wear, femoral component loosening, excessive bone loss in revisions, delamination of the hydroxyapatite coating, and femoral stem fracture at the impaction site on the neck of the stem. Venable, Stuck, and Beach introduced a hemiarthroplasty made of Vitallium (1939), which faced issues due to acetabular wear [2]. Phillip Wiles stainless steel MoM THA (1938), Thompson-Moore implant (1950), the McKee-Farrar THA, and Mittelmeier Prosthesis with axial “Weightbearing Ribs” (1974) demonstrated femoral component loosening secondary to inadequate fixation [1,2,3,11,14,18]. Early fully coated cylindrical femoral stems (1973) exhibited excellent osteointegration but were associated with massive bone loss in the revision setting [11]. The fully porous coated Anatomic Modular Locking (AML) stem (1977) demonstrated significant metaphyseal bone loss secondary to stress shielding [11]. Some early cementless femoral stem designs (1986) experienced the delamination of the hydroxyapatite coating and subsequent loosening [11]. Some cementless M30NW stainless steel femoral stems with impaction sites (2003) broke at the impaction site on the neck of the stem [11]. The distribution of mechanical failures in hip arthroplasty can be found in Table 3.

Mechanical failures in shoulder arthroplasty include instability, glenoid- and humeral-sided loosening, component breakage, component impingement, and scapular fractures. Nonconstrained prostheses such as the Neer I (1953) hemiarthroplasty, the Neer II anatomic total shoulder arthroplasty (TSA, 1974), and Gerard’s reverse shoulder arthroplasty (RSA, 1973) developed instability with high rates of proximal humeral head migration [4,5]. In 1978, Buechel released a “floating fulcrum” RSA with dual mobility that reported high rates of instability [4]. The smaller size of the glenosphere of the Mark II RSA by Dr. Neer (1977) led to a constrained design that resulted in limited mobility and a high rate of glenoid loosening [5,19]. Modifications made for the Mark III (1981) added axial rotation to the humeral stem to facilitate motion, but this led to an increased rate of loosening and problems with fixation of the scapula [4,5,19]. Fixed-fulcrum systems, such as the Averill 3 by Dr. Neer (1970–1972) and Fenlin’s RSA (1975), intended to leverage the mechanical advantage of the deltoid muscle. However, these implants were plagued with implant breakage, loosening, and instability [4,17]. Additionally, constrained prostheses such as the Beddow and Elloy Liverpool RSA (1969), the Stanmore constrained TSA (1972), the Leeds and Reeves RSA (1972), and the Kessel prosthesis (1985) all reported unacceptably high rates of glenoid loosening [5,6,17,20,21]. Michael’s Reese total shoulder (MRTS) described by Post in 1975 reported cases of humeral neck breakage due to the undersizing of the prosthesis [17,21]. The second iteration of the MRTS (1977), Dr. Cofield’s metal-backed glenoid (1980), the second generation modular TSA (1985), and the trispherical system by Gristina and Webb (1978) all had design problems that led to inadequate fixation and failure of the glenoid component [4,5,17]. Monoblock tantalum metal-backed glenoids (2013) commonly failed due to fracture of the glenoid peg-base plate junction [22]. In 1985, Grammont’s “Trompette” RSA was originally implanted and eventually led to high rates of notching, loosening, and breakage of the glenoid component [5,17,22]. In 1991, the Delta III reverse shoulder was released but encountered complications such as instability, scapula fracture, and glenoid loosening [5,23,24]. The third generation of Grammont’s design (1994) implemented a diaphyseal stem on the humeral component, but medial impingement and instability were persistent causes of failure [5,19,25]. Mechanical failures in shoulder arthroplasty are described in Table 4.

#### 3.2.3. Technical Failures

Technical failures include excessive reaming leading to weakened cortical bone, implantation without adequate initial fixation with cement or press-fit surfaces, component undersizing leading to inadequate fixation and fracture, and over lateralization of stems leading to accelerated bearing surface wear. The Rizzoli stem (1977) required excessive reaming, resulting in weakened cortical bone and subsequent failure due to fracture [11]. Early modular taper fluted titanium femoral stems (1982) exhibited late loosening when implanted without cement [11]. The Birmingham hip resurfacing (1997) commonly failed because of femoral neck fractures [26]. The distribution of technical failures in hip arthroplasty can be found in Table 5.

Technical failures in shoulder arthroplasty include infection secondary to inadequate sterilization techniques, tuberosity osteotomies, implantations that require excessive bone resection, and tendon resections for exposure. Notably, Dr. Péan’s first shoulder arthroplasty in 1893 and later König’s ivory prosthesis in 1914 both primarily failed due to infection [6]. The Mark I (1973) had a large anatomical glenoid that required the excessive resection of bone stock and did not allow reattachment of the rotator cuff, leading to the proximal migration of the humeral head with superior impingement and loosening of the glenoid [4,17]. Similarly, due to its design, the Bickel implant (1977) required the removal of a significant amount of bone that resulted in a high rate of failures due to loosening, fractures, and persistent pain [5]. Implantation of the Swanson bipolar hemiarthroplasty (1975) required an osteotomy of the greater tuberosity that led to superior humeral head migration, instability, and ultimately, poor results in terms of recovery of function [4,27]. In 1977, Maza’s nonconstrained total shoulder required a posterior approach and resection of the supraspinatus tendon, and subsequently had poor outcomes, instability, and glenoid loosening [17]. Technical failures in shoulder arthroplasty are listed in Table 6.

## 4. Discussion

Surgical techniques, implant materials, and the understanding of joint biomechanics have significantly advanced since the inception of arthroplasty over 140 years ago as a treatment for debilitating joint diseases. However, ongoing innovation to mitigate common causes of failure underscores the importance of learning from historical missteps. Effective communication among arthroplasty designers is crucial as it ensures the exchange of expertise and insights, akin to how Dr. Charnley’s collaboration with dentists informed his decision to utilize PMMA bone cement [18]. In the current era of orthopedics, implant designers often operate in isolated silos within their subspecialties. Lack of cross-disciplinary communication regarding historical failures and current insights into joint biomechanics, techniques, and materials may result in oversights and the repetition of failures evident in other subspecialized fields. In total hip arthroplasty, many of the failure modes have been addressed with improvements in implant design, biomaterials, and surgical technique.

Material failures that were seen with the use of ivory, glass, PTFE, polyethylene, stainless steel, polyacetal, ceramics, and MoM bearing surfaces were decreased with the use of inert materials with favorable wear properties and low fracture rates [1,2]. Present-day femoral stems utilize stainless steel, cobalt–chromium alloys, and titanium alloys. Although the poor biocompatibility of stainless steel has decreased its use in THA, titanium (Ti-6Al-4V) or cobalt (CoCrMo) alloys have become the most predominant metals used in THA [28]. Moreover, titanium alloys allow for bony ingrowth, making the use of uncemented implants more predictable to provide more durable fixation. Additionally, wear resistance has been improved with the introduction of highly crosslinked molecular weight (HXLE) and ceramic bearings that are being used more frequently in THA [14,25,28]. Implant fracture rates have been decreased with the use of stronger metal alloys such as the ones mentioned above and zirconia-toughened alumina (ZTA) [14,28].

In total shoulder arthroplasty, the improvement in biomaterials was accelerated by some of the failures seen in total hip arthroplasty such as those in the case of the Judet brothers adopting acrylic in hips in 1948 and later Richards, Krueger, and deAnquin doing the same, leading to similar failure modes. The use of ivory, acrylic, and Vitallium have been replaced by more resistant materials that have decreased hardware-related complications [29]. Similarly, shoulder arthroplasty has utilized these biomaterials as in hip arthroplasty, and in doing so, failures that were seen in early total hip designs have been avoided. Currently, humeral stems consist of combinations of metals with titanium (Ti-6Al-4V) or cobalt (CoCrMo), with cobalt being preferable because of improved wear resistance [29]. Similar to hip arthroplasty, bearing surfaces used in shoulder arthroplasty include polyethylene (UHMWPE and highly crosslinked HXLPE), ceramic, and pyrolytic carbon (PyC) [29,30,31]. Ceramic bearings, although widely used in hip arthroplasty, have been a challenge to implement in shoulder arthroplasty. Currently, the only ceramic bearing shoulder implant system on the market in the United States has a warning due to fracture at the coupling [29,30]. The wear properties of pyrolytic carbon have led to its increased popularity and have shown promising results in the hand and elbow [29,30]. The most popular bearing surface configuration consists of cobalt chrome on UHMWPE [29,30].

Mechanical failures such as acetabular wear, femoral component loosening, stress shielding, and stem fracture have all been reduced with advancements in implant design and patient selection. Previously observed acetabular erosions in the setting of hemiarthroplasty have been presently combated with acetabular component implantation, also known as total hip arthroplasty [1]. Initial advancements in stem fixation were attributed to Dr. Charnley’s introduction of PMMA. However, high rates of aseptic loosening due to “cement disease” and “particle disease” set the groundwork for modern uncemented stems with different fixation methods [8]. Femoral component loosening is now less frequent due to improved fixation methods such as refined cementing techniques including preparation under vacuum and the application of lower viscosity under pressure [32]. Additionally, stem stability has improved with the utilization of varied stem geometry such as tapered, fluted, and wedged stems [33]. The shape of the stem decides the pressurization of the cement during insertion and for rotational stability. Understanding this has led to the development of successful cementless stems [34,35,36]. Additionally, the advent of porous coating and press-fit technique after appropriate canal preparation has made uncemented fixation gain popularity. Stress shielding was combated with greater metaphyseal fixation by upsizing the proximal body of the implant, converting a fully porous stems to metaphyseal coating only, using materials with a modulus of elasticity closest to bone, matching the geometry of the stem to that of the femoral canal, and decreasing the length of stems [8,33,37].

Mechanical failures in shoulder arthroplasty have decreased because of iteratively improved implant designs that were driven by applied biomechanical data. Currently, surgeons prevent instability by addressing patient-specific variations in glenoid size and version, glenoid lateralization, humeral head height, soft tissue balancing, and rotator cuff integrity [38]. Glenoid loosening has been addressed by cementing the glenoid component, the introduction of the baseplate and the inlay design in 1985, the use of a central peg and peripheral screw fixation, and the use of augmentation in patients with bone loss [4,17,22,31,39]. Humeral stem loosening has been combated with the use of cement, porous coating to improve press-fit fixation, variations in stem size, stemless humeral implants, inlay versus onlay models, and most recently, the use of convertible platforms to facilitate conversion between a TSA and an RSA in the revision setting [4,31,40]. Unlike the weight bearing hip joint, load distribution across the shoulder is primarily concentrated in the proximal aspect of the humerus. This load distribution in the shoulder leads to the ability to achieve fixation in the humeral metaphysis as opposed to the diaphysis [41]. This has led to the development of different proximal stem geometries and cylindrical stems that engage the endosteal canal to achieve appropriate alignment and fixation [41,42]. Moreover, stem diameter, modularity, and geometry have been shown to be important features to increase fixation and rotational stability in both shoulder and hip arthroplasties [34,35,36,43,44,45]. However, due to the different load distributions across the shoulder, component breakage has different causes when compared to hip arthroplasty. The mechanical breakage of components that was observed mainly in constrained prostheses has ushered in implant designs with less constraints. Component impingement, particularly scapular notching, has decreased because of a reduced neck shaft angle in RSA and by achieving adequate glenoid lateralization and positioning [46,47]. Scapular fractures in RTSA are becoming less frequent by improving bone health, by correcting glenoid baseplate screw length, and by avoiding excessive deltoid tension [48,49]. The introduction of reverse shoulder arthroplasty led to decreased mechanical complications seen in the past, but component geometry is still evolving. The more common “anatomic style reverses” have led to a lower rate of mechanical failures and the improved preservation of functions [50].

Improvements in surgical techniques have minimized technical failures. Practices such as avoiding excessive reaming component undersizing and accurate component positioning have led to a decrease in failures. These advancements have led to a reduction in issues such as component loosening, periprosthetic fracture, and the accelerated wear of bearing surfaces [3,11,26].

Technical failures in shoulder arthroplasty have been reduced by the better understanding of surgical site infections, avoiding osteotomies and excessive bone resection, and favoring muscle-sparing approaches [51,52]. Instability has been mitigated with component upsizing and increasing offset. However, achieving the ideal balance to avoid increased joint reactive forces that will accelerate wear rates is still evolving [53]. Greater tuberosity osteotomies, particularly in the fracture setting, have been associated with lower functional results and more postoperative complications [54]. Currently, the subscapularis sparing technique has been shown to reduce instability risk, but is more technically complex, and limited exposure may lead to the incorrect fixation of the humeral head [52]. These advancements have led to alternatives to address instability and a reduction in infection rates, rotator cuff failures, and implant failures.

The benefit of shoulder arthroplasty emerging behind the developments in total hip replacement surgery is demonstrated by several innovations in the shoulder that were developed using evidence from failed total hip. For example, impingement-free range of motion, a feature that led to some failures of total hips, was then minimized with the introduction of larger femoral head implants. This was then expanded in RSA with the introduction of lateralized glenosphere to increase impingement-free ROM. Additionally, the reduced amount of material failures in shoulders compared to hips, as evident by the 10% failures in shoulders compared to the 48% failures in hips, validates this benefit.

The future benefit of hip arthroplasty and shoulder arthroplasty surgeons collaborating could be the use of preop planning software that may lead to improvements in placing implants to maximize fixation and optimize function such as impingement-free ROM in both total shoulder and hip arthroplasties. Moreover, the introduction of the reverse hip emulating the concept of the reverse total shoulder shows the inspiration taken from other subspecialties [55,56]. Although there has been little utilization of this type of prosthesis, the development of the reverse hip demonstrates that collective research between specialties can increase treatment options for recurrent complications and ultimately improve patient outcomes [55,56,57].

This review has several limitations. One limitation is the oversimplification of each modality of implant failure. The authors acknowledge that most implant failures are multifactorial in nature. Another limitation is that the categorization of the failures themselves is subjective to the authors and are not precise but more of a generalization. An additional limitation is that failed implants were mostly extracted from historical articles; thus, the information available is limited by what is reported in those publications. It should also be known that the named failures were not meant to target any brand but rather state a failure that was described in the literature. However, a strength of our article is that this is the first time a classification of failure modes has been proposed. By grouping similar failure mechanisms, it simplifies the way surgeons and implant designers can address issues in a collaborative form with other subspecialties in orthopedics.

## 5. Conclusions

Our research underscores the significance of understanding past total hip and shoulder arthroplasty failures. Despite the shared similarities between hip and shoulder arthroplasty, it is apparent that the implementation of innovations has been delayed in terms of transitioning from one field to the other. This delay is presumably attributed to designers’ lack of awareness regarding both the shortcomings and successes in other subspecialties. Although there are inherent anatomic and biomechanical differences in the hip and shoulder joints, valuable insights from previous successes and failures in one field can inform and enhance design considerations in the other. In today’s age, implant designers rarely seek to collaborate with those from other orthopedic subspecialities. This emphasizes the need for a more integrated approach in the development of implant designs that considers the lessons learned from various orthopedic disciplines.

## Figures and Tables

**Table 1:** Material failures in hip arthroplasty with the reported year of release and a brief description of failure mechanism.

Hip Implant	Year	Description of Failure Mode
Ivory arthroplasty by Gluck	1880	Foreign body reaction and infection
Smith-Petersen mold/cup arthroplasty	1923	Breakage of glass during gait, and other molds lacked inert and durable properties
Judet acrylic THA ^1^	1948	Prosthesis wear, fracture of the prosthesis, and bone absorption.
Charnley PTFE ^2^ THA	1955	PTFE wear and failure
Charnley MoP ^3^ THA	1970	Polyethylene wear particles led to aseptic loosening
Polyacetal femoral heads	1971	Failed screw or glue fixation led to femoral head/neck fracture
Screw or glue fixation of femoral head	1972	Failed screw or glue fixation led to femoral head/neck fracture
Stainless steel heads replaced by aluminum oxide ceramic heads	1974	Wear particle production
Ceramic-on-ceramic (alumina and zirconia)	1985	Chipping, squeaking, and fracture
316 L stainless steel femoral stems	1983	Fatigue fracture of stem and aseptic loosening
Third generation, large diameter MoM bearing surfaces	1988	Metallosis, hypersensitivity reactions, and aseptic loosening

^1^ Total hip arthroplasty, ^2^ polytetrafluoroethylene, and ^3^ metal-on-polyethylene.

**Table 2:** Material failures in shoulder arthroplasty with the reported year of release and a brief description of failure mechanism.

Shoulder Implant	Year	Description of Failure Mode
MacAusland polyamide prosthesis	1950s	Lack of durability, wear particle production, and foreign body reactions
Judet acrylic shoulder prosthesis	1952	Lack of durability, wear particle production, and foreign body reactions
Richards, Krueger, and deAnquin acrylic TSA	1955	Lack of durability, wear particle production, and foreign body reactions

**Table 3:** Mechanical failures in hip arthroplasty with the reported year of release and a brief description of failure mechanism.

Hip Implant	Year	Description of Failure Mode
Phillip Wiles MoM ^1^ THA ^2^	1938	High stress concentration and femoral loosening
Venable, Stuck, and Beach HA ^3^ made of Vitallium	1939	Acetabular wear
Thompson-Moore implant	1950	Femoral component loosening
McKee-Farrar MoM THA	1950	Femoral component loosening
Fully coated cylindrical Lord stem	1973	Excellent osteointegration led to massive bone loss in revision setting
Mittelmeier “weightbearing ribs” prosthesis	1974	Inadequate fixation led to femoral component loosening
Anatomic Modular Locking (AML) fully coated stem	1977	Stress shielding led to large metaphyseal bone loss in revisions
Cementless stems with hydroxyapatite coating	1986	Delamination of the hydroxyapatite coating caused implant loosening
Cementless M30NW stainless steel femoral stem with impaction site	2003	Broke at impaction site on neck of stem

^1^ Metal-on-Metal, ^2^ Total Hip Arthroplasty, ^3^ Hemiarthroplasty.

**Table 4:** Mechanical failures in shoulder arthroplasty with the reported year of release and a brief description of failure mechanism.

Shoulder Implant	Year	Description of Failure Mode
Neer I hemiarthroplasty	1953	Instability with humeral head migration
Beddow and Elloy Liverpool Shoulder	1969	Glenoid loosening
Neer Averill 3 “fixed fulcrum” prosthesis	1970–1972	Implant breakage, loosening, and instability
Stanmore constrained TSA ^1^	1972	Implant breakage and glenoid loosening
Leeds/Reeves RSA ^2^ with divergent screws	1972	Glenoid loosening
Gerard reverse prosthesis	1973	Instability with proximal humeral head migration and acromial erosion
Neer II anatomic total shoulder	1974	Instability with proximal humeral head migration
Fenlin RSA fixed-fulcrum system	1975	Implant breakage, loosening, and instability
Michael Reese total shoulder (MRTS)—first iteration, constrained reverse	1975	Humeral neck breakage due to undersizing
Neer Mark II	1977	Limited mobility and glenoid loosening
MRTS second iteration	1977	Constrained model led to impingement and glenoid fixation issues
Buechel “floating fulcrum” RSA	1978	Instability (allowed supraphysiologic ROM)
Gristina and Webb’s trispherical system	1978	Inadequate fixation and failure of glenoid component
Cofield’s TSA metal-backed glenoid	1980	Inadequate fixation led to glenoid loosening
Neer Mark III	1981	High rates of component loosening and problems with scapula fixation
Kessel prosthesis	1985	Glenoid loosening
Second generation modular TSAs	1985	Inadequate fixation led to glenoid loosening
Grammont’s Trompette: glenoid baseplate with press-fit central peg	1985	Notching, loosening, and breakage of the glenoid component
Delta III reverse	1991	Instability, scapula fracture, and glenoid loosening
Grammont III variation	1994	Medial impingement and instability
Monoblock tantalum metal-backed glenoid TSA	2013	Fracture of the glenoid peg-base plate junction
Lateralized RSA		

^1^ Total shoulder arthroplasty and ^2^ reverse shoulder arthroplasty.

**Table 5:** Technical failures in hip arthroplasty with the reported year of release and a brief description of failure mechanism.

Hip Implant	Year	Description of Failure Mode
Rizzoli stem	1977	Elliptical proximal body and round distal required excessive reaming and weakened cortical bone
Modular taper fluted titanium femoral stem	1982	Late loosening when implanted without cement
Birmingham hip resurfacing	1997	Femoral neck fracture

**Table 6:** Technical failures in shoulder arthroplasty with the reported year of release and a brief description of failure mechanism.

Shoulder Implant	Year	Description of Failure Mode
Jules Emile Péan	1893	Chronic infection
König ivory prosthesis	1914	Infection and septic loosening
Neer Mark I	1973	Size of prosthesis prevented rotator cuff attachment, superior migration, pain, and dysfunction
Swanson bipolar HA ^1^	1975	Greater tuberosity osteotomy led to superior migration, instability, and poor functional results
Bickel implant	1977	Extensive bone removal for implantation led to high complication rates
Mazas nonconstrained TSA ^2^	1977	Resected supraspinatus led to poor outcomes, instability, and glenoid loosening

^1^ Hemiarthroplasty and ^2^ total shoulder arthroplasty.
